# Supplementary material for: Genome-wide methylation data mirror ancestry information
Source: Epigenetics Chromatin. 2017 Jan 3;10:1. doi: 10.1186/s13072-016-0108-y (PMC5267476; doi:10.1186/s13072-016-0108-y)
Supplement: Supplementary file 1 — Additional file 1. Supplementary note for more details. [file 13072_2016_108_MOESM1_ESM.pdf]

Supplementary Information For:

Genome-wide methylation data mirror ancestry information

Elior Rahmani, Liat Shenhav, Regev Schweiger, Paul Yousefi, Karen Huen, Brenda Eskenazi, Celeste Eng, Scott Huntsman, Donglei Hu, Joshua Galanter, Sam Oh, Melanie Waldenberger, Konstantin Strauch, Harald Grallert, Thomas Meitinger, Christian Gieger, Nina Holland, Esteban Burchard, Noah Zaitlen, and Eran Halperin.

Results Summary						
Data Set	Meth PCs	Adj PCs	Adj PCs II	Barfield et al.	EPISTRUCTURE	Measurement
GALA II	$R^2 = 0.01$	$R^2 = 0.83$	$R^2 = 0.70$	$R^2 = 0.02$	$R^2 = 0.83$	Genotype-based PC 1
	$R^2 = 0.02$	$R^2 = 0.32$	$R^2 = 0.27$	$R^2 = 0.03$	$R^2 = 0.32$	EU fraction
	$R^2 = 0.01$	$R^2 = 0.81$	$R^2 = 0.69$	$R^2 = 0.03$	$R^2 = 0.81$	NA fraction
	$R^2 < 0.01$	$R^2 = 0.79$	$R^2 = 0.67$	$R^2 < 0.01$	$R^2 = 0.78$	AF fraction
CHAMACOS	$R^2 = 0.04$	$R^2 = 0.15$	$R^2 = 0.14$	$R^2 = 0.05$	$R^2 = 0.38$	Genotype-based PC 1
	$R^2 = 0.03$	$R^2 = 0.11$	$R^2 = 0.08$	$R^2 = 0.01$	$R^2 = 0.46$	EU fraction
	$R^2 = 0.04$	$R^2 = 0.14$	$R^2 = 0.11$	$R^2 = 0.01$	$R^2 = 0.60$	NA fraction
	$R^2 = 0.01$	$R^2 = 0.01$	$R^2 = 0.01$	$R^2 = 0.01$	$R^2 = 0.06$	AF fraction
GALA II 450K-specific CpGs excluded	$R^2 = 0.01$	$R^2 = 0.83$	$R^2 = 0.70$	$R^2 = 0.04$	$R^2 = 0.82$	Genotype-based PC 1
	$R^2 = 0.02$	$R^2 = 0.32$	$R^2 = 0.28$	$R^2 = 0.03$	$R^2 = 0.31$	EU fraction
	$R^2 = 0.01$	$R^2 = 0.81$	$R^2 = 0.69$	$R^2 = 0.04$	$R^2 = 0.80$	NA fraction
	$R^2 = 0.01$	$R^2 = 0.55$	$R^2 = 0.46$	$R^2 = 0.03$	$R^2 = 0.55$	AF fraction
CHAMACOS 450K-specific CpGs excluded	$R^2 = 0.04$	$R^2 = 0.15$	$R^2 = 0.14$	$R^2 = 0.05$	$R^2 = 0.33$	Genotype-based PC 1
	$R^2 = 0.03$	$R^2 = 0.11$	$R^2 = 0.08$	$R^2 = 0.04$	$R^2 = 0.45$	EU fraction
	$R^2 = 0.04$	$R^2 = 0.14$	$R^2 = 0.11$	$R^2 = 0.04$	$R^2 = 0.58$	NA fraction
	$R^2 = 0.01$	$R^2 = 0.01$	$R^2 = 0.01$	$R^2 = 0.04$	$R^2 = 0.08$	AF fraction

Table S1: Summary of the results in the GALA II data set and in the CHAMACOS data set. In the first part of the table, squared linear correlations were measured between several measurements of ancestry information and linear predictors using the first two PCs of the data (Meth PCs), the first two PCs after adjusting the data for cell type composition (Adj PCs), the first two PCs after adjusting the data for cell type composition and excluding probes containing SNPs from the data (Adj PCs II), the first two PCs when considering only CpGs in close proximity to SNPs (Barfield et al.) and the first two EPISTRUCTURE PCs. The second part of the table presents the results of the same experiments, only after excluding all the CpGs of the 450K array that were not included in the EPIC methylation array.

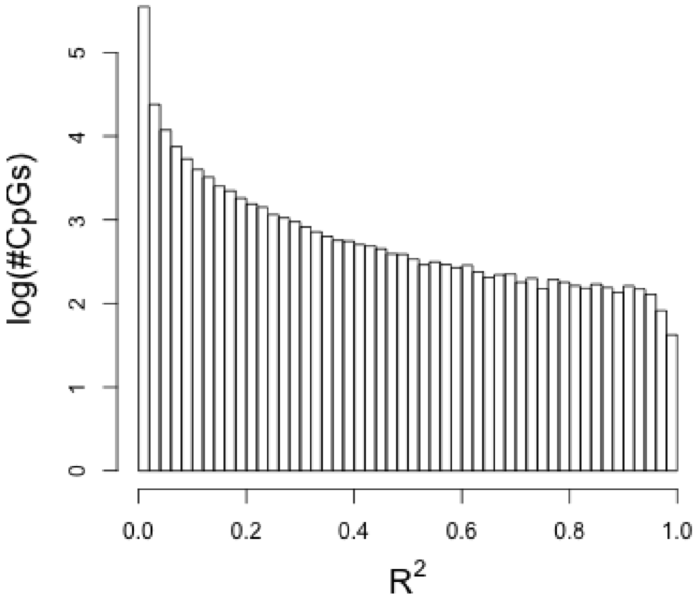

Figure S1: Correlation of methylation sites with cis-SNPs in the KORA data set. An R^2 score was calculated for each CpG available in the data from cis-SNPs (see Methods). The results are presented in a log scaled histograms, showing that in most of the CpGs only a small portion of the variance can be explained by cis-SNPs.

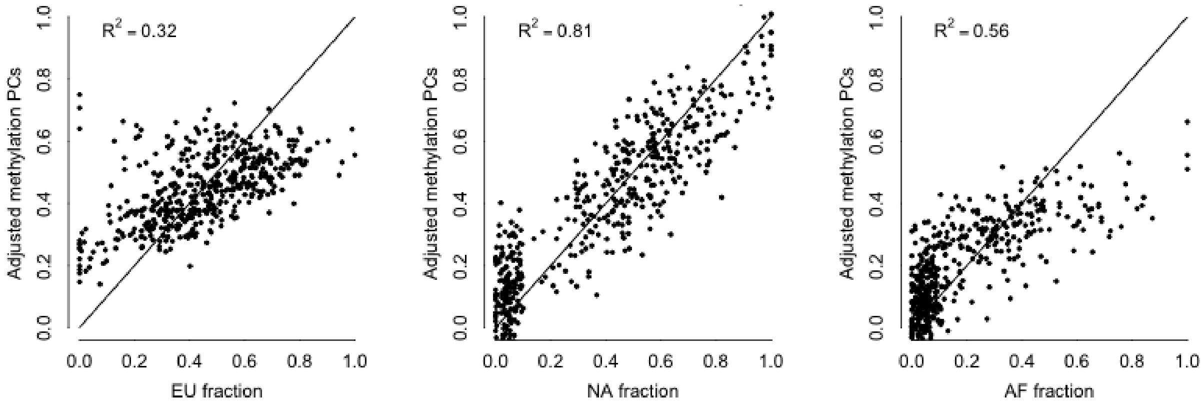

Figure S2: Capturing ancestry fraction estimates in the GALA II data using EPISTRUCTURE. Presented are linear predictors of European (EU), Native-American (NA) and African (AF) fraction estimates of the individuals in the data using the first two EPISTRUCTURE PCs.

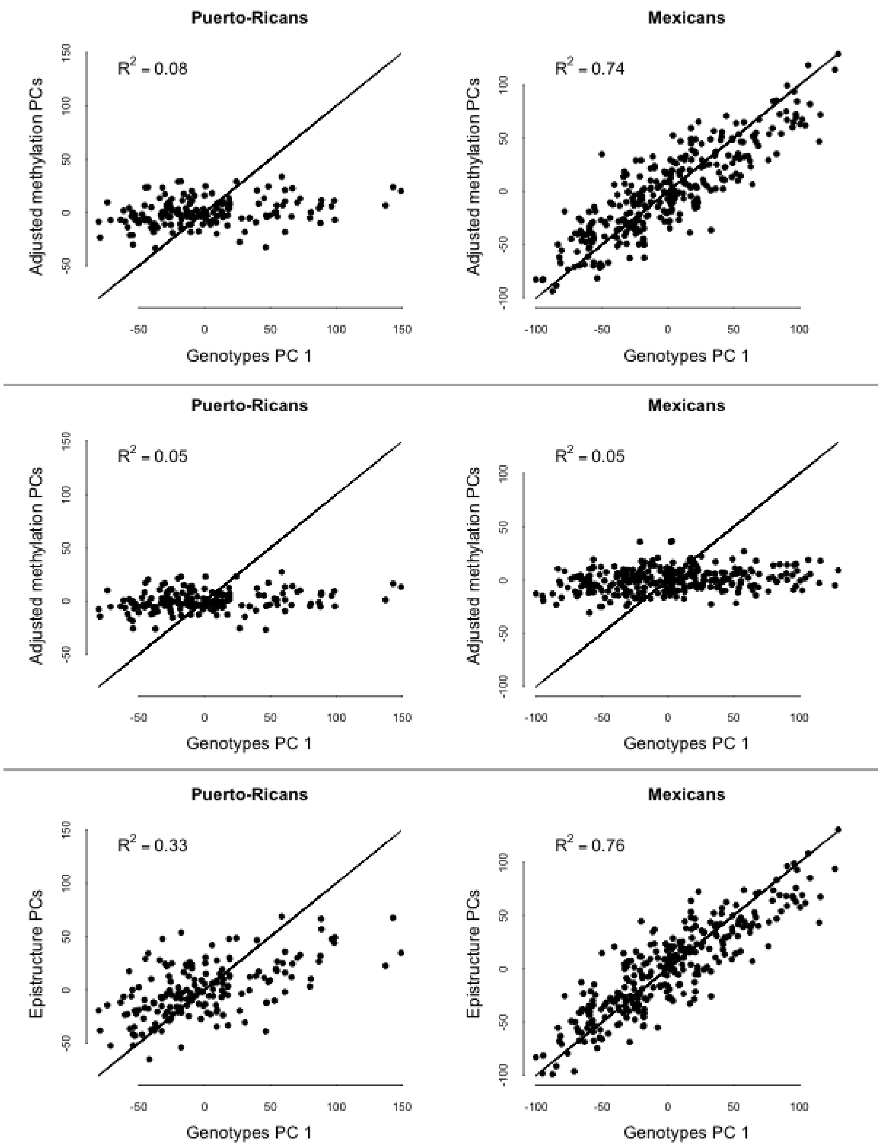

Figure S3: Capturing ancestry information in the GALA II data from Puerto-Rican (PR) individuals and from Mexican (MX) individuals separately. Presented are linear predictors of the first genotype-based PC using the first two methylation PCs computed from each subpopulation separately after adjusting the data for cell composition, before and after excluding probes containing SNPs from the data (top and middle panels, respectively) and using the first two EPISTRUCTURE PCs (bottom panel).

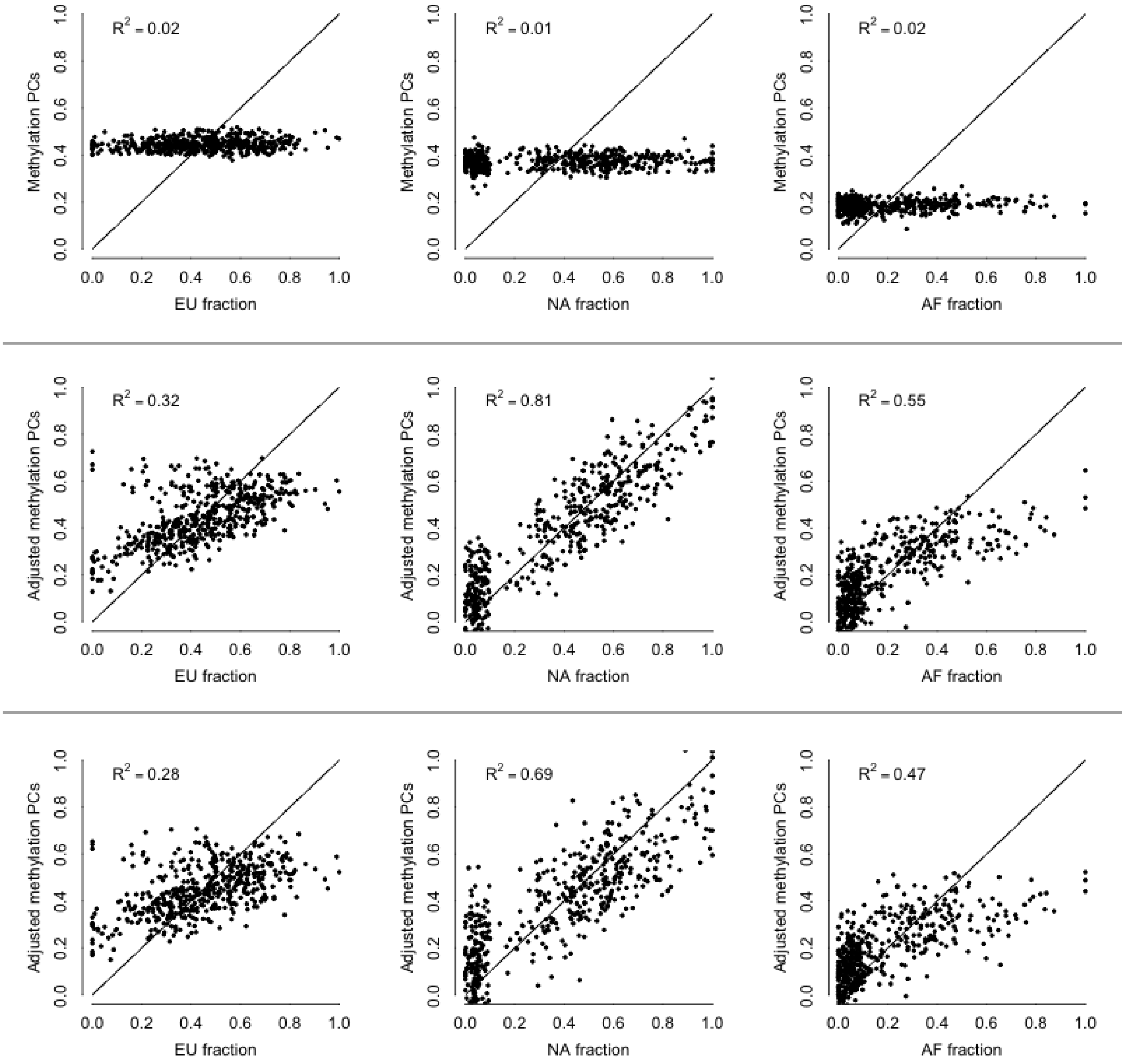

Figure S4: Capturing ancestry fraction estimates in the GALA II data. Presented are linear predictors of European (EU), Native-American (NA) and African (AF) fraction estimates of the individuals in the data using the first two methylation PCs of the data (top panel), the first two PCs after adjusting the data for cell composition (adjusted methylation PCs; middle panel) and using the adjusted methylation PCs after excluding from the data all probes containing SNPs (bottom panel).

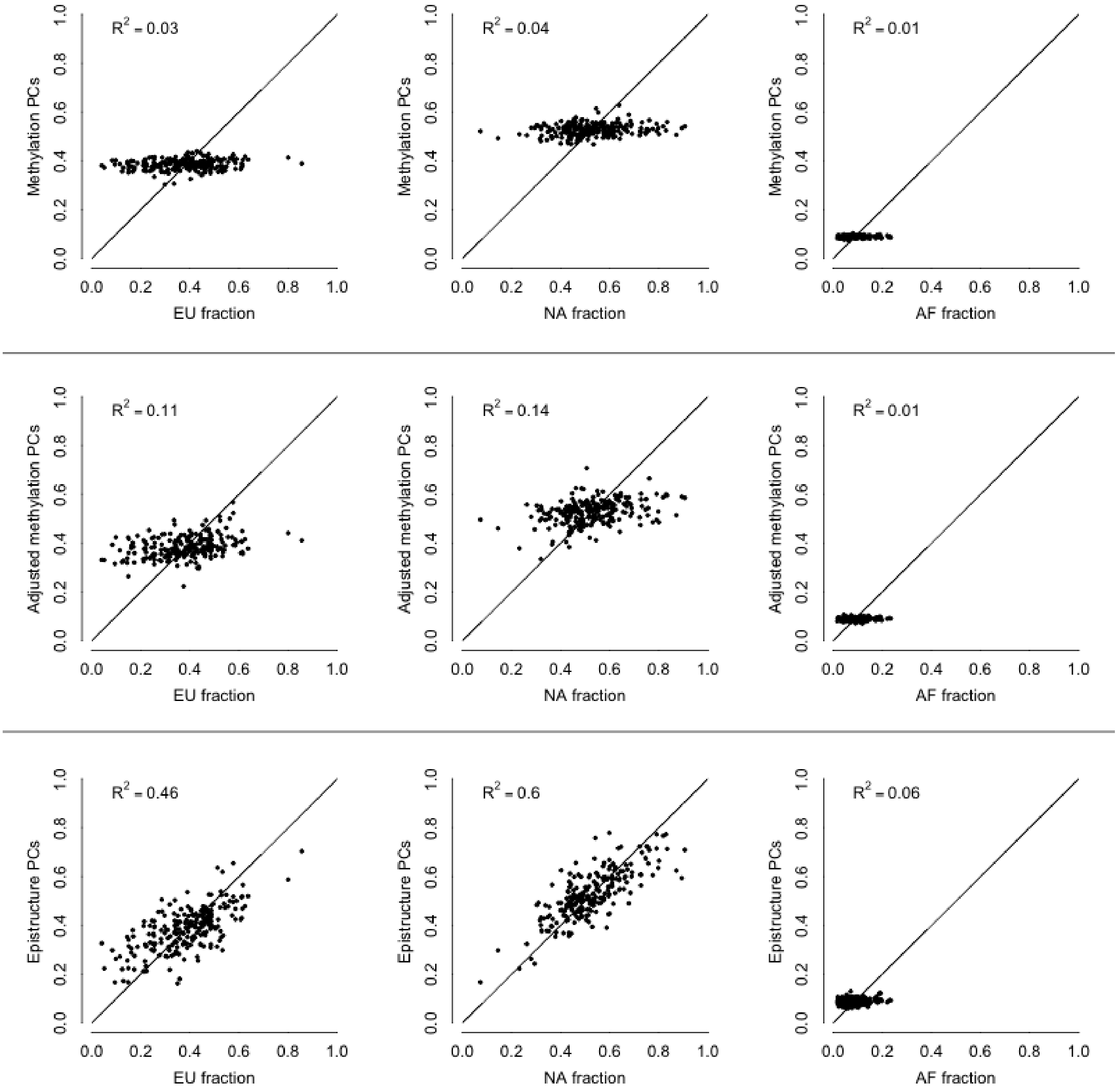

Figure S5: Capturing ancestry fraction estimates in the CHAMACOS data set. Presented are linear predictors of European (EU), Native-American (NA) and African (AF) fraction estimates of the individuals in the data using the first two methylation PCs (top panel), the first two PCs after adjusting the data for cell type composition (adjusted methylation PCs; middle panel) and using the first two EPISTRUCTURE PCs (bottom panel). The methylation PCs in this experiment were computed without excluding probes containing SNPs from the data.

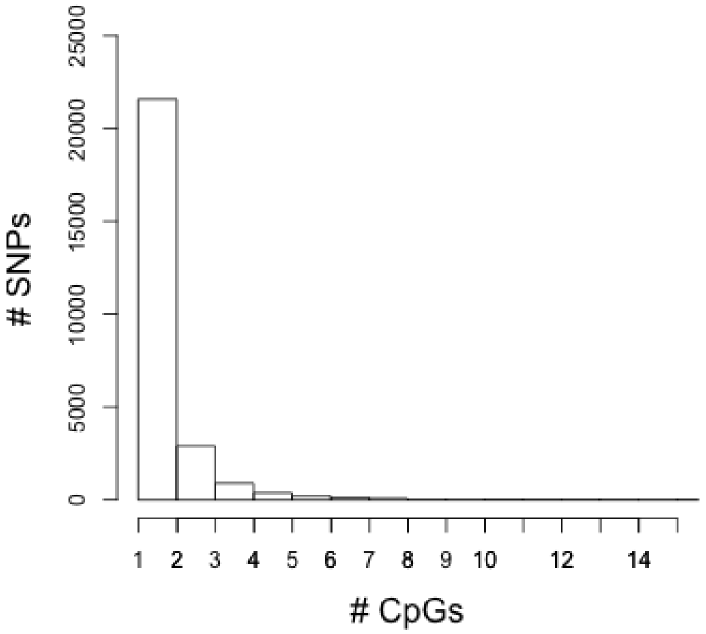

Figure S6: Most of the available SNPs used in creating the reference list of genetically-informative CpGs were found to be predictors of no more than one CpG in the reference list. Only 26,244 out of the available SNPs in KORA (657,103) were used in the prediction of the CpGs that were included in the reference list. Out of these sites 82.2% were found to be predictors of only one CpG and 93.3% were found to be predictors of at most two CpGs.

Supplementary note

PCA is well-known to efficiently capture ancestry information when applied to genotypes data [1]. In this supplementary note we show why applying PCA on CpGs that are linear combinations of SNPs is expected to capture population structure as well. The algorithm of EPISTRUCTURE can be divided into two main steps. First, a reference list of genetically-informative methylation sites is compiled from a group of CpGs, each found to be well approximated by its cis-located SNPs. Second, given a new methylation data set, the first several PCs of the data are calculated only from the sites that were included in the reference list. The reason for applying PCA in the second part of the algorithm is motivated by the success of PCA to capture ancestry information in genotyping data. In the case of genotyping data coming from different populations, the first several PCs capture population structure by highlighting groups of individuals differing at the level of allele frequencies. Given an $n \times s$ centered genotyping data matrix G of s SNPs collected from n individuals, the generative model underlying PCA assumes:

$$G = ZW + \Sigma \tag{1}$$
$$\Sigma_j \sim MVN\left(0, \tau^2 I_n\right)$$

where Z is an $n \times k$ matrix representing k-dimensional latent structure of the ancestry information for each individual and W is a $k \times s$ matrix representing ancestry-specific differences in allele frequencies for each SNP. Σ is an $n \times s$ error term, typically assumed to have independent entries (that is, no relatedness between the n individuals and independence between the SNPs).

Any methylation site can be modeled as a linear function of SNPs and additional error term, and therefore the methylation level of a specific site in a given individual can be approximated to some extent using merely the individual's SNPs. Formally, given an $n \times m$ centered methylation data matrix O of m methylation sites coming from the same n individuals in G, we can describe O_j, the j-th column of O as:

$$O_j = GB_j + E_j \tag{2}$$
$$E_j \sim MVN\left(0, \sigma_j^2\right)$$

where B_j is an $s \times 1$ coefficients vector of the linear model and E_j is an $n \times 1$ error term. In particular, methylation site j that cannot be even partially explained by SNPs will have a corresponding B_j vector of

only zeros. In the first step of the EPISTRUCTURE algorithm we find a group of methylation sites which can be well explained by their cis-located SNPs. Restricting the data matrix O to be consisted only of such methylation sites increases the signal-to-noise ratio in the data.

Plugging (1) into (2) we get

$$\begin{aligned} O_j &= (ZW + \Sigma)B_j + E_j && (3) \\ &= ZWBj + \Sigma B_j + E_j && (4) \end{aligned}$$

where ΣB_j and E_j are normally distributed as before. This model can be equivalently described as follows:

$$O_j \sim MVN\left(ZWB_j, \left(B_j^t B_j \tau^2 + \sigma_j^2\right) I_n\right) \quad (5)$$

Under this formulation there is a dependency between every two methylation sites. However, based on previous reports showing clear predominance of associations between CpGs and cis-located SNPs over trans-located SNPs [2–4], we assume that only cis-located SNPs are informative for explaining a given methylation site. As a result, B is expected to be very sparse with values concentrated around the diagonal, assuming the SNPs and CpGs are ordered by physical position. In particular, every two distant methylation sites are independent. In our case the matrix B was estimated from the KORA data for which both genotyping and methylation levels were available. We observed that the vast majority of the rows in the estimated matrix are sparse and only rarely have more than one non-zero entry (Supplementary Figure S6). The main reason for this is the fact that we consider only a sparse set of methylation sites from the genome, resulting from the first step of the algorithm in which only sites that can be well approximated by SNPs are selected. Therefore, we neglect the theoretical dependency between close sites and assume no dependency between any of the columns in B. Now, the model can be summarized as:

$$O_j \sim MVN(Z\tilde{W}_j, \psi_j^2 I_n) \quad (6)$$

where $\tilde{W}_j = WB_j$ and we are interested in extracting Z, the latent ancestry information structure of the individuals in the data. The maximum likelihood solution to the model in (3) is given by factor analysis, and the first k factors can be used as estimates of the latent population structure Z. In practice, factor analysis iteratively scales each site and the first iteration is equivalent to PCA after standardization of each

of the sites. Empirically, applying more than one iteration did not improve the performance, therefore, in the second step of the EPISTRUCTURE algorithm we suggest to perform a standardized PCA and to consider the first k PCs as the estimate of the population structure.

References

[1] Alkes L Price, Nick J Patterson, Robert M Plenge, Michael E Weinblatt, Nancy A Shadick, and David Reich. Principal components analysis corrects for stratification in genome-wide association studies. *Nature genetics*, 38(8):904–909, 2006.

[2] Jordana T Bell, Athma A Pai, Joseph K Pickrell, Daniel J Gaffney, Roger Pique-Regi, Jacob F Degner, Yoav Gilad, Jonathan K Pritchard, et al. Dna methylation patterns associate with genetic and gene expression variation in hapmap cell lines. *Genome Biol*, 12(1):R10, 2011.

[3] Dandan Zhang, Lijun Cheng, Judith A Badner, Chao Chen, Qi Chen, Wei Luo, David W Craig, Margot Redman, Elliot S Gershon, and Chunyu Liu. Genetic control of individual differences in gene-specific methylation in human brain. *The American Journal of Human Genetics*, 86(3):411–419, 2010.

[4] Degui Zhi, Stella Aslibekyan, Marguerite R Irvin, Steven A Claas, Ingrid B Borecki, Jose M Ordovas, Devin M Absher, and Donna K Arnett. Snps located at cpg sites modulate genome-epigenome interaction. *Epigenetics*, 8(8):802–806, 2013.
